# Hafnium-Substituted Wells–Dawson Polyoxometalate as a High-Performance Contrast Agent for Transmission Electron Microscopy of Biological Ultrastructure

**DOI:** 10.3390/ijms27125523

**Published:** 2026-06-18

**Authors:** Aleksandra Milosavljević, Mila Ćetković, Tamara Kravić-Stevović, Marko Stojanović, Mirjana B. Čolović, Nada D. Savić, Tatjana N. Parac-Vogt, Danijela Krstić

**Affiliations:** 1 Institute of Histology and Embryology, Faculty of Medicine, University of Belgrade, 11000 Belgrade, Serbia; aleksandra.milosavljevic@med.bg.ac.rs (A.M.); mila.cetkovic-milisavljevic@med.bg.ac.rs (M.Ć.); tamara.kravic-stevovic@med.bg.ac.rs (T.K.-S.); 2 Department of Pharmacology, Clinical Pharmacology and Toxicology, Faculty of Medicine, University of Belgrade, 11000 Belgrade, Serbia; marko.stojanovic@med.bg.ac.rs; 3 Department of Physical Chemistry, “Vinča” Institute of Nuclear Sciences—National Institute of the Republic of Serbia, University of Belgrade, 11351 Belgrade, Serbia; colovicm@vin.bg.ac.rs; 4 Department of Chemistry, KU Leuven, Celestijnenlaan 200F, 3001 Leuven, Belgium; nada.savic@uvsq.fr; 5 Institute of Medical Chemistry, Faculty of Medicine, University of Belgrade, 11000 Belgrade, Serbia

**Keywords:** Wells-Dawson polyoxometalates, transmission electron microscopy, contrast agent, hafnium, ultrastructural imaging, signal-to-noise ratio, kidney tissue

## Abstract

Transmission electron microscopy (TEM) is a key tool for ultrastructural analysis, yet its performance critically depends on contrast agents, many of which are constrained by toxicity and regulatory concerns. In this study, Wells–Dawson (WD)-type polyoxometalates (POMs), including Parent WD-POM, Lacunary WD-POM, Zr-WD 1:2 POM, and Hf-WD 1:2 POM, were investigated as alternative contrast agents for TEM imaging of rat kidney tissue. Among the tested compounds, Hf-WD 1:2 POM provided the most consistent contrast enhancement, enabling clear visualization of subcellular structures with minimal background interference and no detectable aggregation. Its contrast performance depended on both concentration and incubation time, with higher concentration (0.01 mol/L) and prolonged incubation (24 h) yielding increased signal-to-noise ratio (SNR), although SNR alone did not fully reflect image quality. In comparison with conventional staining agents (uranyl acetate and Uranyless), Hf-WD 1:2 POM achieved significantly improved contrast effectiveness without inducing detectable tissue damage. These findings identify Hf-WD 1:2 POM as a promising non-radioactive alternative for TEM imaging and support the potential of POMs as versatile platforms for the development of advanced contrast agents.

## 1. Introduction

Transmission Electron Microscopy (TEM) is a crucial imaging technique for visualizing subcellular structures at high magnifications, widely utilized in biological, medical and materials science research [1]. In TEM, an electron beam passes through a thin specimen, and variations in electron density within the sample create contrast in the resultant image [1,2]. An adequate contrast agent is of high importance to selectively enhance the electron density of specific tissue components, thereby improving the overall image quality. The most used contrast agents in TEM are heavy metal salts, such as uranyl acetate (UAc) and lead citrate, which bind to biological molecules, increase the scattering of electrons, and enhance contrast [3,4,5]. While effective, these agents have key limitations such as toxicity, instability, and radioactivity, which has led to increasing interest in alternative contrast agents for TEM [4,5]. In this regard, polyoxometalates emerge as an attractive alternative.

Polyoxometalates (POMs) are a diverse class of anionic clusters consisting of transition-metal centres (typically W, Mo, or V in their highest oxidation states) connected through oxo ligands [6,7]. These discrete clusters are known for their unique and highly tenable structural characteristics, including their variable molecular architectures, solubility in different solvents, and well-defined redox properties [6,7,8,9]. Due to their versatile properties and structural diversity, POMs have found utility in a wide array of scientific and technological disciplines and have exhibited promising biological activities [8,10,11,12,13,14,15,16,17,18,19]. For instance, their relatively large molecular size and well-defined three-dimensional shape enable them to interact selectively with biological macromolecules [10]. Growing interest in the biological applications of POMs has led to an increasing number of studies aimed at elucidating their functional potential. One particularly active area of investigation is the application of POMs as contrast agents for enhancing the visualization of internal structures in imaging techniques such as magnetic resonance imaging (MRI), computed tomography (CT), and TEM [4,5,12,20,21]. POMs have gained increasing attention in TEM applications owing to several intrinsic advantages, such as high negative charge density, structural adaptability, and excellent stability [8,22,23,24].

**Scheme 1:**
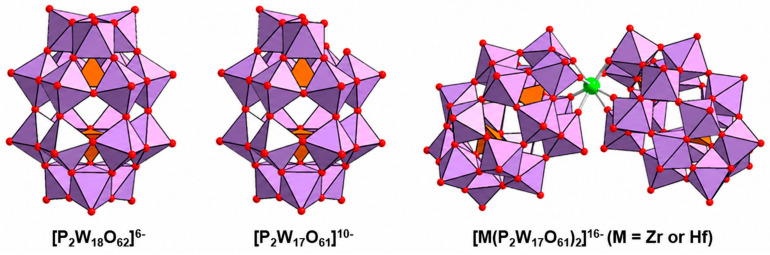
The polyhedral representation of WD POMs (Parent WD—[P_2_W_18_O_62_]^6−^, Lacunary WD—[P_2_W_17_O_62_]^10−^, M-WD 1:2 (M = Zr(IV) or Hf(IV)—[M(P_2_W_17_O_61_)_2_]^16−^). WO_6_ and PO_4_ are represented by violet octahedra and orange tetrahedra, respectively. The green spheres represent Zr(IV) or Hf(IV) ions. Red spheres represent oxygen molecules.

Among the diverse families of POMs, we selected four Wells–Dawson-type (WD) POMs to investigate their potential as contrast agents for TEM: Parent WD-POM, Lacunary WD-POM, Hf-substituted WD POM (Hf-WD 1:2), and Zr-substituted WD POM (Zr-WD 1:2) (Scheme 1).

This class of POMs offers several favourable characteristics: their inherently high electron density supports strong contrast generation, their tunability and well-defined structures allow for chemical functionalization to improve solubility and biological compatibility, and importantly, they are generally associated with comparatively low toxicity profiles, which enhances their suitability for biological imaging applications [25]. The parent Wells–Dawson POM ([P_2_W_18_O_62_]^6−^) represents a structurally robust and highly electron-dense framework composed of 18 tungsten atoms arranged around two phosphate groups [25,26,27,28]. Lacunary POM (α_2_-[P_2_W_17_O_61_]^10−^) is derived from the parent structure by the removal of one metal–oxide unit, creating reactive site that enable the coordination of additional metal centres and further functionalization [25,29,30,31]. The incorporation of transition metals such as hafnium or zirconium into lacunary Wells–Dawson results in the formation of (K_16_[Hf(α_2_-P_2_W_17_O_61_)_2_]·19H_2_O) and (K_15_H[Zr(α_2_-P_2_W_17_O_61_)_2_]·25H_2_O), respectively, further modifying POM properties, including stability, electron density, and potential interactions with biological systems [25,32,33,34,35,36,37,38,39,40,41,42,43,44,45]. According to the available literature, this study represents the first systematic evaluation of hafnium-substituted WD POMs as contrast agents for TEM of biological ultrastructure. Our screening studies identified Hf-WD 1:2 POM as the most promising contrast agent for TEM, and its concentration and incubation time were further optimized to enhance its performance. Furthermore, the performance of Hf-WD 1:2 POM was compared with traditional contrast agents such as UAc and Uranyless to evaluate its potential as a promising contrast agent for TEM imaging. Given the challenges of traditional agents, our research could offer valuable insights into the development of less toxic and effective contrast agents for imaging biological tissues at the ultrastructural level and may contribute to the development of safer and more versatile contrast agents for biological TEM imaging.

## 2. Results

### 2.1. Evaluation of Contrast Performance of Wells-Dawson Polyoxometalates

**Figure 1:**
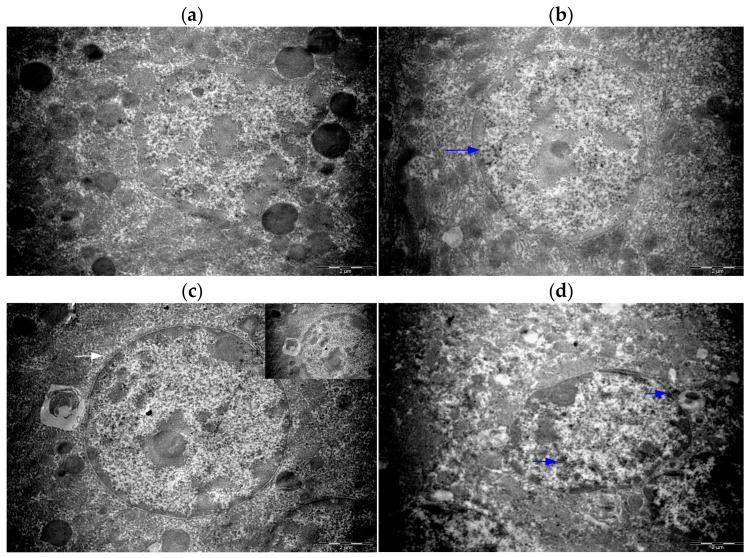
Transmission electron microscopy (TEM) images of rat kidney tissue (7.1 k, scale bar: 2 µm) stained with four different polyoxometalates (0.001 mol/L, 1 min): (**a**) Parent WD-POM; (**b**) Lacunary WD-POM; (**c**) Hf-WD 1:2 POM; and (**d**) Zr-WD 1:2 POM. Hf-WD 1:2 POM provided the most consistent structural delineation (white arrow), while Lacunary and Zr-WD 1:2 POM showed evidence of agent accumulation (blue arrows). The white arrow in panel (**c**) indicates the region of interest shown at higher magnification (8.9 k) in the inset.

The contrast performance of four polyoxometalates (Parent WD-POM, Lacunary WD-POM, Hf-WD 1:2 POM, and Zr-WD 1:2 POM) was evaluated in rat kidney tissue sections following contrast staining at a concentration of 0.001 mol/L for 1 min. Representative TEM micrographs are shown in Figure 1. All tested compounds enabled visualization of subcellular structures and demonstrated varying levels of contrast enhancement. Parent WD-POM provided moderate contrast, allowing identification of nuclei and mitochondria, although with limited sharpness and increased background noise (SNR = 12.06). Lacunary WD-POM improved structural delineation (SNR = 14.09), but partial loss of clarity was observed due to background interference and occasional formation of nanoparticle-like aggregates, likely reflecting its lower solubility. In contrast, Hf-WD 1:2 POM enabled superior visualization of intracellular structures, including clear discrimination between cytoplasm, membranes, and organelles such as mitochondria and rough endoplasmic reticulum (SNR = 12.9). Importantly, no evidence of compound accumulation was observed. Although Zr-WD 1:2 POM exhibited the highest SNR value (14.2), the corresponding images showed reduced structural clarity and visible accumulation of the contrast agent. Despite comparable or higher SNR values observed for some compounds, Hf-WD 1:2 POM provided the most consistent and interpretable contrast enhancement and was therefore selected for further analysis.

### 2.2. Optimization of Hf-WD 1:2 POM Concentration and Incubation Time

**Figure 2:**
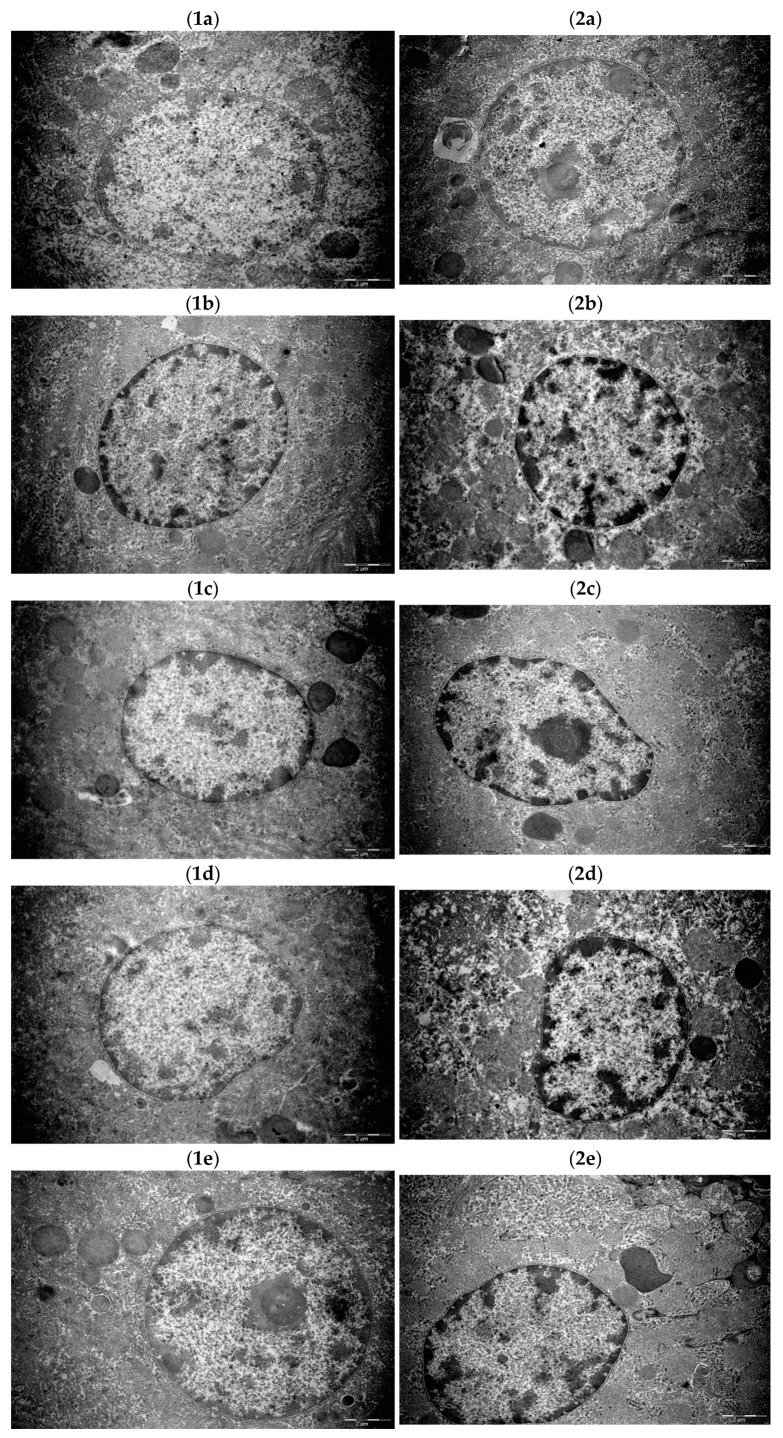

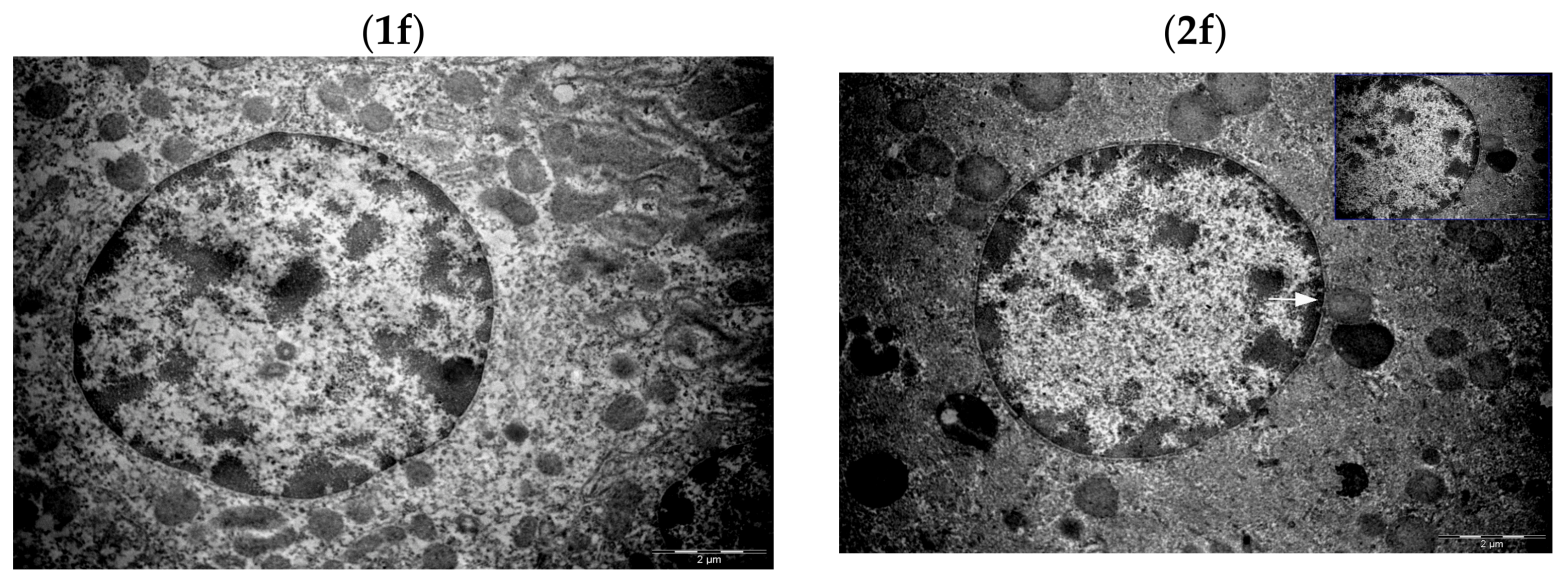
Transmission electron microscopy (TEM) images of rat kidney tissue (7.1 k, scale bar: 2 µm) stained with Hf-WD 1:2 POM at two concentrations and varying incubation times: (**1**) 0.001 mol/L and (**2**) 0.01 mol/L; (**a**) 1 min; (**b**) 15 min; (**c**) 20 min; (**d**) 30 min; (**e**) 1 h; and (**f**) 24 h. Contrast enhancement increased with both concentration and incubation time, with the highest signal-to-noise ratio (SNR) values observed at prolonged incubation (24 h). Based on the overall evaluation of contrast and structural preservation, staining at 0.01 mol/L and 24 h was selected for subsequent analyses. The white arrow in panel (**2f**) indicates the region of interest shown at higher magnification (8.9 k) in the inset.

The effect of Hf-WD 1:2 POM concentration and incubation time on contrast enhancement was evaluated using two concentrations (0.001 and 0.01 mol/L) at six incubation times (1 min, 15 min, 20 min, 30 min, 1 h, and 24 h). Representative TEM images are shown in Figure 2. At a concentration of 0.001 mol/L, contrast enhancement showed a gradual increase with prolonged incubation time. Initial staining at 1 min resulted in moderate contrast (SNR = 12.9), with limited structural definition. Extension of incubation to 15 and 20 min led to slight improvements in organelle visibility, accompanied by a modest increase in SNR (12.89 and 13.26, respectively). Further increase to 30 min resulted in improved delineation of cellular structures (SNR = 13.4). However, prolonged incubation at 1 h led to partial loss of image clarity, likely due to staining saturation (SNR = 11.2). The highest SNR value at this concentration was observed after 24 h (14.68), indicating increased signal intensity. At a higher concentration (0.01 mol/L), overall contrast was consistently improved across all incubation times. At 1 min, contrast was higher compared to the lower concentration (SNR = 13.44). Increasing incubation time to 15 and 20 min further enhanced image clarity and structural definition (SNR = 14.87 and 15.15, respectively). At 30 min and 1 h, SNR values remained high (14.88 and 15.12), indicating a plateau in contrast enhancement. The highest SNR value (15.57) was achieved after 24 h, corresponding to pronounced contrast and clear visualization of subcellular structures. Overall, contrast enhancement depends on both concentration and incubation time, with higher concentration (0.01 mol/L) consistently yielding improved results. The highest SNR values were observed at prolonged incubation (24 h), although variations in structural clarity across conditions indicate that SNR alone does not fully reflect image quality.

### 2.3. Comparative Evaluation of Hf-WD 1:2 POM and Conventional Contrast Agents (Uranyl Acetate and Uranyless) in TEM Imaging

**Figure 3:**
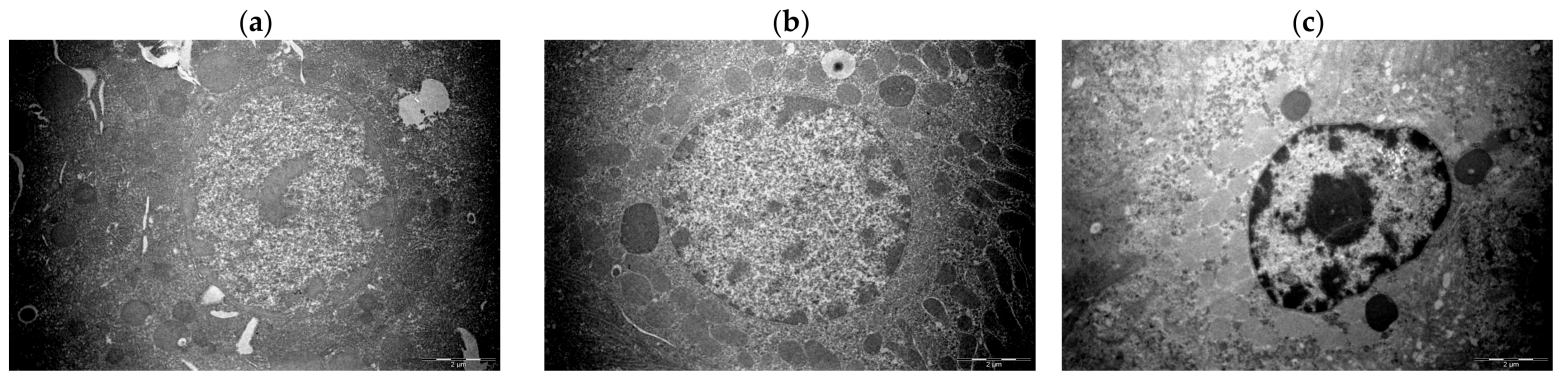
Transmission electron microscopy (TEM) images of rat kidney tissue (7.1 k, scale bar: 2 µm) stained with: (**a**) uranyl acetate; (**b**) Uranyless; and (**c**) Hf-WD 1:2 POM (0.01 mol/L, 24 h). Hf-WD 1:2 POM (0.01 mol/L, 24 h) enabled adequate contrast compared to conventional agents.

**Figure 4:**
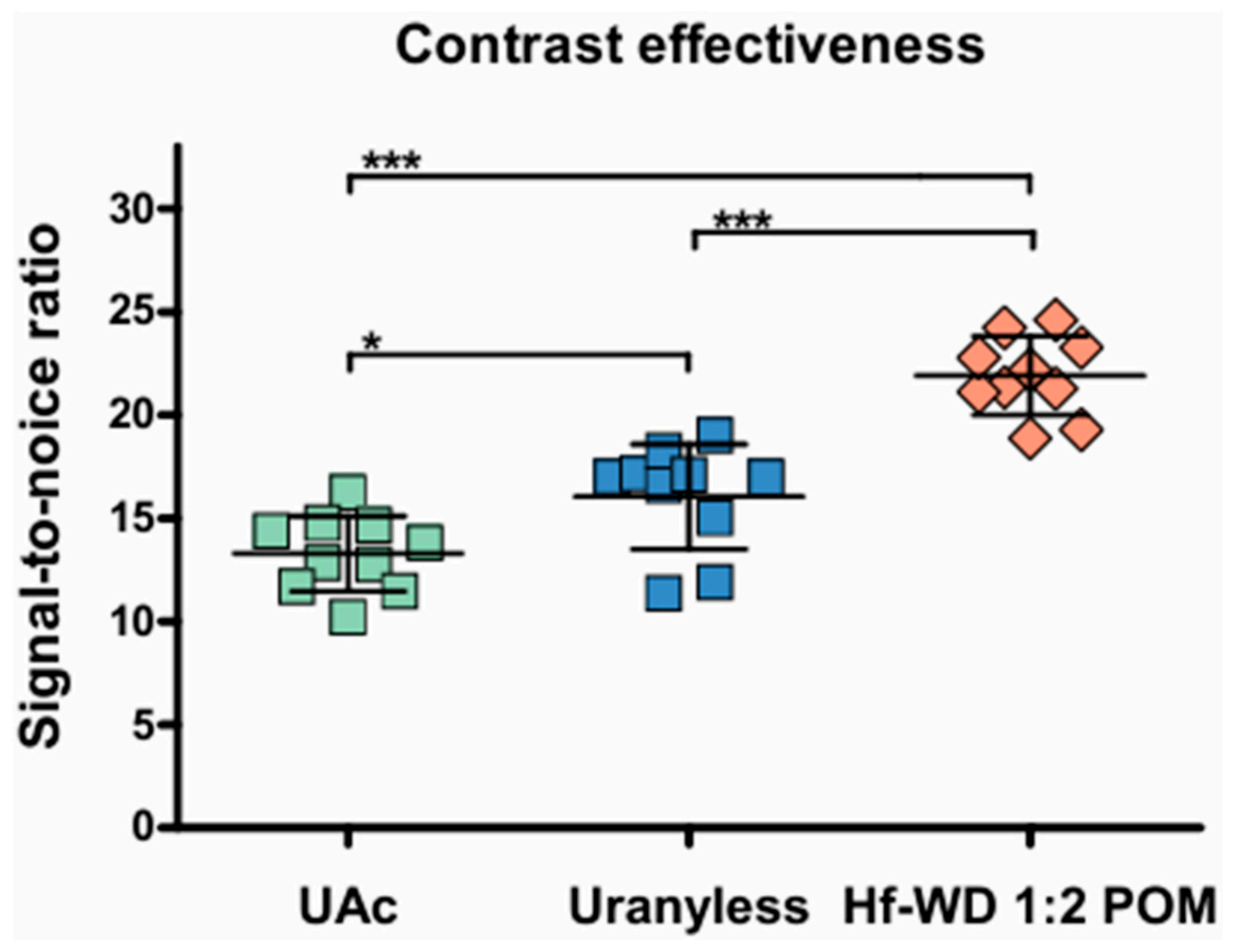
Quantitative analysis of signal-to-noise ratio (SNR) values obtained from TEM images of kidney tissue stained with uranyl acetate, Uranyless, and Hf-WD 1:2 POM (0.01 mol/L, 24 h). Data are presented as mean ± SD. The significance values: *** *p* < 0.001; * *p* < 0.05.

The performance of Hf-WD 1:2 POM (0.01 mol/L, 24 h) was compared with conventional staining agents, uranyl acetate (UAc) and Uranyless, in kidney tissue samples fixed with glutaraldehyde without osmium tetroxide, to observe the single effects of the investigated contrasts. When compared with the traditional agents, uranyl acetate and Uranyless, Hf-WD 1:2 POM showed markedly higher contrast at 0.01 mol/L and 24 h incubation (Figure 3), providing significant contrast effectiveness and minimal background noise. The average SNR was 21.9, with individual measurements reaching up to 24.61. In comparison, uranyl acetate produced moderate contrast (SNR = 13.29), but with increased background noise and reduced visibility of fine structural details. Uranyless yielded slightly improved background uniformity compared to uranyl acetate but lower overall structural definition (SNR = 16.04). Quantitative analysis confirmed significant differences between the tested agents and demonstrated that under the evaluated conditions, Hf-WD 1:2 POM achieved significantly higher SNR values compared to both uranyl acetate and Uranyless (*p* < 0.001) (Figure 4). Collectively, these results indicate that Hf-WD 1:2 POM (0.01 mol/L, 24 h) could substitute conventional contrast agents in TEM imaging, providing enhanced contrast and reduced background interference.

## 3. Discussion

In this study, we evaluated structurally modified Wells–Dawson-type POMs as contrast agents for transmission electron microscopy (TEM) of biological tissues, with the aim of improving contrast quality, biocompatibility, and practical applicability compared to conventional staining agents such as uranyl acetate and commercially available alternatives like Uranyless. To the best of our knowledge, this is the first study systematically investigating hafnium-substituted WD POMs as TEM contrast agents for biological tissue ultrastructure while simultaneously evaluating staining optimization parameters and directly comparing their performance with uranyl acetate and Uranyless. The obtained results demonstrate that structural modifications of the Wells–Dawson framework significantly influence contrast performance, with the hafnium-substituted derivative (Hf-WD 1:2 POM) showing the most favourable balance between contrast enhancement, structural clarity and stability.

The comparison of the four investigated POM variants revealed that their contrast efficiency is strongly dependent on their structural and chemical properties. Parent WD-POM, as a fully saturated and chemically stable structure, exhibited the lowest contrast enhancement, which can be attributed to its limited reactivity and reduced ability to interact with biological macromolecules. In contrast, the lacunary derivative, characterized by the removal of one {WO_6_} unit, showed improved contrast, likely due to the presence of a reactive defect site that enables stronger interactions with protein and lipid components of the cell, as previously reported for lacunary POM systems [29,31]. However, this increased reactivity was accompanied by reduced solubility and the formation of nanoparticle-like aggregates, which may result in non-uniform staining and imaging artefacts, consistent with earlier observations for similar systems [46].

Further enhancement of contrast was achieved through metal substitution within the lacunary structure. Zr-WD 1:2 POM exhibited slightly higher SNR values under certain conditions, indicating that the incorporation of metal centres contributes to contrast efficiency [37,39,47]. However, its performance was limited by visible accumulation of precipitate and reduced image clarity, suggesting that SNR alone is not sufficient to fully describe contrast quality. In contrast, Hf-WD 1:2 POM demonstrated the most consistent and interpretable contrast enhancement, combining sufficient SNR values with superior structural delineation and homogeneous distribution within the kidney tissue. The improved performance of this compound can be attributed to the high atomic number of hafnium, which enhances electron scattering and facilitates better visualization of subcellular structures [36], as well as to its favourable coordination within the lacunary Wells–Dawson framework.

The influence of concentration and incubation time on staining efficiency further supports the importance of optimizing application parameters. At lower concentration (0.001 mol/L), a gradual increase in SNR with incubation time was observed, indicating time-dependent interaction with tissue components; however, the achieved contrast remained limited. At higher concentration (0.01 mol/L), significantly improved contrast was observed across all time points, with the highest SNR values obtained after prolonged incubation. This behaviour is consistent with diffusion-driven uptake and progressive interaction with intracellular structures such as nuclear membranes, nucleoli, and mitochondria [48]. Importantly, the relationship between incubation time and image quality was not strictly linear, indicating that maximal SNR does not necessarily correspond to optimal structural preservation. Therefore, the selected experimental conditions were based on a balance between contrast enhancement and preservation of ultrastructural integrity rather than on SNR values alone.

Comparison with conventional contrast agents confirmed the advantages of Hf-WD 1:2 POM. The achieved contrast was higher than that obtained with both uranyl acetate and Uranyless. Uranyl acetate, although widely used, presents significant limitations related to toxicity and radioactivity, which complicate its use in routine laboratory practice [49,50]. In addition, its performance may be affected by increased background noise and limited structural resolution, as previously reported [3,5]. Uranyless, developed as a safer alternative, showed improved safety and a more uniform background; however, it did not reach the level of contrast achieved with Hf-WD 1:2 POM. These findings are consistent with previous studies highlighting the potential of metal-based nanoclusters and POM systems as efficient, non-radioactive imaging agents [4,5,13]. As far as we are aware, there are no previous studies evaluating WD POMs for biological ultrastructure visualization by TEM. One of the recent Italian studies [51] reported improved TEM contrast using lanthanide-polyoxometalate formulations, while Sahiro et al. described Preyssler-type polyoxotungstates as promising negative staining reagents for viral TEM imaging [52]. In addition, de Bournonville and the team demonstrated the applicability of hafnium-substituted WD POMs as contrast-enhancing agents for soft tissue micro-CT imaging without significant tissue distortion [33]. Although the aim of this study was to assess the individual effects of the three contrast agents and thereby demonstrate superior contrast performance of Hf-WD 1:2 POM, our results suggest that combining Hf-WD 1:2 POM with osmium tetroxide, as used in the standard TEM preparation protocol, may further improve ultrastructural visualization (Figure 2).

Based on the obtained results, Hf-WD 1:2 POM appears particularly suitable for biological samples requiring enhanced visualization of membrane-rich and organelle-dense regions, such as kidney tubular epithelium. However, its performance in other tissues such as highly mineralized tissues, lipid-rich samples, or specimens with limited permeability remains to be investigated. Additional studies will therefore be required to determine its applicability across different biological models and preparation protocols.

An important requirement for any TEM contrast agent is the preservation of tissue ultrastructure. In the present study, Hf-WD 1:2 POM did not induce detectable structural damage, such as membrane disruption, or cytoplasmic disorganization, even after prolonged incubation, which is consistent with previous findings indicating the relatively low toxicity and good biocompatibility of POMs under physiological conditions [28,29]. These findings suggest that the investigated POM does not significantly alter tissue morphology under the applied experimental conditions. The stability of the compound in aqueous solution and at near-neutral pH further contributes to its suitability for biological applications. The absence of staining-induced artefacts is particularly important for the reliable interpretation of ultrastructural data.

The superior performance of Hf-WD 1:2 POM can be explained by a combination of different physicochemical factors. The high atomic number of hafnium contributes to enhanced electron scattering, directly improving contrast in TEM imaging [36,47]. In addition, the free coordination sites of Hf substituted in the Wells–Dawson structure provides reactive sites that enable stable interactions with biological macromolecules [8,36]. Unlike uranyl acetate, which primarily binds to negatively charged functional groups and may be displaced under certain conditions, Hf-WD 1:2 POM likely forms more stable coordination interactions, although further studies are required to fully elucidate the binding mechanisms. Its larger molecular structure may also contribute to selective localization within specific subcellular compartments. Furthermore, the absence of radioactive properties eliminates concerns related to radiolysis and long-term instability, supporting its use in extended imaging protocols [6,7,8].

The findings of this study also suggest the broader applicability of POM-based contrast agents beyond TEM. Due to their structural tunability and high electron density, compounds such as Hf-WD 1:2 POM may be adapted for use in other imaging modalities, including scanning electron microscopy, X-ray imaging, and correlative imaging approaches. Previous studies have demonstrated the potential of POMs in computed tomography applications [13,20,21], supporting their versatility as imaging agents. Nevertheless, this study has certain limitations, including its evaluation in only a single tissue type and its reliance on SNR as the primary quantitative metric. Although SNR provided a useful quantitative parameter for contrast comparison, image quality in TEM also depends on additional factors including structural preservation, edge definition, staining homogeneity, and background uniformity. Therefore, qualitative ultrastructural assessment was performed in parallel with SNR analysis, since clear visualization of biologically relevant subcellular structures remains one of the most important practical criteria in transmission electron microscopy, despite the partially subjective nature of visual interpretation. The qualitative analysis of the electron micrographs was performed by three experienced microscopists in order to minimize bias, while the analysts were blinded to the identity of the samples during the evaluation process. A direct quantitative comparison of SNR values with previously published studies is currently difficult because most reports evaluating alternative TEM contrast agents rely predominantly on qualitative ultrastructural assessment rather than standardized quantitative image analysis. Differences in sample preparation, fixation protocols, staining conditions, microscope settings, and image acquisition parameters further complicate comparisons between studies. In this context, the present work contributes additional quantitative insight by combining qualitative ultrastructural evaluation with SNR-based analysis of contrast performance under controlled experimental conditions. Future studies should therefore investigate the performance of Hf-WD 1:2 POM across different biological samples and incorporate additional metrics to enable a more comprehensive evaluation of image quality.

## 4. Materials and Methods

### 4.1. Chemicals

Wells-Dawson POMs: (Parent WD POM, Lacunary WD POM, Hf-WD 1:2 POM, and Zr-WD 1:2 POM) were synthesized and characterized as described in the literature [24,44]. Uranyl acetate, Uranyless, and other chemicals were purchased from Sigma-Aldrich (St. Louis, MO, USA) and prepared according to the manufacturer’s instructions [53,54].

### 4.2. Ethical Approval

The methodological procedures used in this study received approval from the Ethical Commission for Experimental Animal Welfare Protection at the Faculty of Medicine, University of Belgrade (N°14729/2-2025). All procedures adhered to the guidelines set by the European Convention for the Protection of Vertebrate Animals Used for Experimental and Other Scientific Purposes and full compliance with Good Laboratory Practice (GLP) was ensured.

### 4.3. Experimental Animals

In this experiment we used a single, healthy, 5-week-old, male *Wistar albino* rat weighing 190 g. All animal procedures were carried out in accordance with institutional guidelines for the care and use of laboratory animals. The study was conducted under controlled surroundings, with a 12 h light/dark cycle, a temperature of 23 ± 2 °C and a humidity of 55 ± 10%. The chosen rat was housed in a polycarbonate cage with three other rats, fed with standard food for rodents and had access to tap water.

### 4.4. Tissue Preparation for TEM

Prior to tissue extraction, the rat was anesthetized. After the kidneys were harvested, 5–6 small kidney tissue pieces (1 mm^3^) were immediately placed in eppendorf tubes filled with 3% glutaraldehyde in cacodylate buffer for 24 h. That way, the primary fixation of the kidney tissue was done. The fixed kidney tissues were then post-fixed with 1% osmium tetroxide for 1.5 h to enhance tissue contrast. A part of the kidney tissue pieces was fixed only with 3% glutaraldehyde in cacodylate buffer without post-fixation with osmium tetroxide. Then, the tissue sections from both fixation groups were washed in distilled water and dehydrated in an increasing-concentration alcohol series (50% for 5 min, 70% for 30 min at 4 °C, 96% for 30 min at 4 °C and 100% three times for 10 min). A further procedure was followed by infiltration of tissue in propylene oxide, then in a mixture of propylene oxide and epoxy resin medium, and finally in epoxy resin medium (Sigma-Aldrich, 45345). After polymerization at 60 °C for 48 h, the kidney tissues were sectioned from the resin blocks into ultrathin slices (approximately 70 nm thick) with a Leica ultramicrotome (Leica Microsystems, Wetzlar, Germany) operating with a diamond knife. Thin sections were mounted onto 300-mesh copper grids (Sigma-Aldrich, G4901), and as with all previous steps, prepared for the following staining procedures with different substances (Parent WD POM, Lacunary WD POM, Hf-WD 1:2 POM, Zr-WD 1:2 POM, uranyl acetate and Uranyless) for examination on a transmission electron microscope (Morgagni 268D, FEI, Hillsboro, OR, USA).

### 4.5. Contrast Agents Preparation and Application

The POMs were dissolved in distilled water. All solutions of the contrast agents were prepared fresh before use and applied to the tissue sections. A drop of the agent was placed on parafilm, and then the copper grid with sample was placed on the drop. After different incubation times, every grid was washed with deionized water, and that way, they were ready for TEM examination.

In the first part of the experiment, the tissue sections were stained with 4 separate stains: Parent WD POM, Lacunary WD POM, Hf-WD 1:2 POM, and Zr-WD 1:2 POM. Each tissue section was stained with one droplet of stain for 1 min at room temperature. The concentration of each stain was 0.001 mol/L.

In the second part of the experiment, we had two groups of tissue sections stained with Hf-WD 1:2 POM in two different concentrations—the concentration of the first Hf-WD 1:2 POM solution was 0.001 mol/L, and the concentration of the second one was 0.01 mol/L. Each group was stained with a single droplet of stain of a certain concentration in 6 different time intervals: 1 min, 15 min, 20 min, 30 min, 1 h, and 24 h. Tissue sections from both parts of the experiment were fixed both with glutaraldehyde and osmium tetroxide.

In the third part of the experiment, we used tissue sections fixed only in glutaraldehyde. Here, we stained tissue sections with 3 different stains: Hf-WD 1:2 POM at chosen concentration and incubation time (concentration of 0.01 mol/L for 24 h), and uranyl acetate and Uranyless at standard concentrations for 1 min. All three sections were stained at room temperature.

For the uranyl acetate and Uranyless treatments, standard solutions were prepared, respectively, as recommended by the literature [51,52]. Uranyl acetate was prepared at a concentration of 4% in methanol solution, and Uranyless was used following the manufacturer’s instructions.

### 4.6. TEM Imaging and Analysis

Following staining, the ultrathin tissue sections mounted on 300 mesh-copper grids were examined under a transmission electron microscope (Morgagni 268D, FEI, Hillsboro, OR). The acceleration voltage of the TEM was 80 kV and the objective aperture was 20 µm. Images were captured with a 16-bit slow-scan CCD camera at 7.1 k nominal magnification to assess the contrast and resolution achieved with each contrast agent. The quality of the images was evaluated based on clarity, contrast, and the resolution of subcellular structures, particularly the nuclei of the kidney tubular cells.

### 4.7. Contrast Quantification: Signal-to-Noise Ratio (SNR)

The TEM contrast was quantified using ImageJ software (version 1.54, National Institutes of Health, Bethesda, MD, USA) [55], where pixel intensity values were recorded and compared across the different contrast agents and incubation conditions. For TEM contrast ratio calculations, we used signal-to-noise ratio (SNR) to compare and evaluate the contrast effectiveness in electron microscopy. SNR is an essential parameter in imaging methods, such as electron microscopy, as it measures the strength of the desired signal in relation to background noise. In electron microscopy, the signal refers to the actual details or information captured, like variations in electron density that create contrast in the image. The term noise encompasses any interference that does not represent the sample, including electronic noise from detectors, shot noise, and thermal noise. A higher SNR signifies a clearer, more distinct image with improved contrast, as the meaningful signal greatly surpasses the noise. By calculating the SNR for images captured under various conditions or with different microscopy techniques, one can objectively compare which setup delivers superior contrast. When evaluating different samples or preparation methods, SNR provides an unbiased metric for determining which approach yields images with the best contrast. Also, high SNR contributes to sharper images, where edges and boundaries are well-defined, while low SNR often results in blurred images, with edges appearing fuzzy and less distinct.

To calculate SNR in electron microscopy, we used the formula:

$$SNR=\frac{\sigma noise}{\mu signal}$$

where *μsignal* is the mean signal level and *σnoise* is the standard deviation of the noise. We captured 10 photomicrographs per each contrast agent treatment. The ratio from every ten-photomicrograph group was averaged and plotted with corresponding standard deviation.

### 4.8. Statistical Analysis

All data analyses were performed using GraphPad Prism (version 10, Graph Pad Software, San Diego, CA, USA). Data were analyzed using one-way analysis of variance (ANOVA) followed by Tukey’s post hoc test to compare differences between groups. Graphs were constructed as scatter plots showing individual values with mean ± SD. Statistical significance was considered when the *p*-value was less than 0.05.

## 5. Conclusions

In this study, structurally modified WD POMs were systematically evaluated as alternative contrast agents for the TEM of biological tissues. Among the investigated compounds, Hf-WD 1:2 POM demonstrated the most favourable overall performance, combining improved contrast enhancement, good tissue compatibility, and stable staining behaviour. Optimization experiments further showed that staining efficiency depended on both concentration and incubation time, with 0.01 mol/L and 24 h incubation providing the highest contrast performance under the applied experimental conditions. In comparison with conventional uranyl acetate and Uranyless staining, Hf-WD 1:2 POM enabled enhanced visualization of subcellular structures while avoiding the disadvantages associated with radioactive uranyl-based reagents. Although additional studies involving different tissue types, biological replicates, and complementary quantitative image analysis approaches are still necessary, the obtained findings support the potential of hafnium-substituted WD POMs as promising non-radioactive contrast agents for biological TEM imaging and the further development of advanced ultrastructural imaging applications.

## Data Availability

The original contributions presented in this study are included in the article. Further inquiries can be directed to the corresponding authors.
